# Extreme ‘gender critical’ views will alienate many gender dysphoric patients

**DOI:** 10.1192/bjb.2021.76

**Published:** 2021-10

**Authors:** Neil David MacFarlane

**Affiliations:** Independent Psychiatrist, Royal College of Psychiatrists, UK. Email: nmacfa01@mail.bbk.ac.uk

The authors provide a welcome update of evidence and reasoning for the ‘gender critical’ position, and the problems with the ‘gender affirming’ position that the Royal College of Psychiatrists adopted in 2018. Their argument might have been even stronger if it had included an account of recent complaints about pressures against free enquiry and expression in this area.^1^ Such pressures may partly account for the paucity of published gender critical clinical and scientific research. Neither did the authors mention the professional misconduct of a ‘gender affirming’ consultant psychiatrist in a London teaching hospital, which was brought to the public's attention in 2007 by the campaigning journalist Julie Bindel.^2^ Or, specifically, the influence of the pharmaceutical industry, although that was implied in at least one of the references they cited.^3^ However, some of their reasoning appears to lean towards an extreme gender critical position, which is inconsistent with mainstream psychiatric practice. They write that ‘there is little to no convincing evidence to support fundamental differences between the brains of females and males’. But a lack of reliable neurobiological pathology is true of most psychiatric disorders, for which psychiatrists routinely prescribe drugs and other physical treatments, quite often coercively. Again, ‘As a pure subjective experience, [gender identity] may be overwhelming and powerful but is also unverifiable and unfalsifiable’. Psychiatric disorders are mainly diagnosed on the basis of what patients report about their ‘subjective experience’, so the requirement that transgender patients must provide substantial additional ‘verification’ of their experiences also suggests that the authors have adopted a double standard. Do they propose that patients with depression or post-traumatic stress disorder demonstrate that their problems are ‘falsifiable’ before they can receive treatment? The authors attempt to distance themselves from ‘conversion therapy’, but many gender dysphoric patients will not find their arguments convincing. They claim ‘there is little evidence’ that transgender conversion therapy ‘is taking place in the UK’, but the 2018 National LGBT Survey found that 13% of UK trans respondents ‘had been offered’ conversion therapy, compared with 7% of ‘cisgender’ respondents.^4^ Conversion therapy for homosexuality is closely associated with psychoanalysis.^5^ The American Psychiatric Association removed homosexuality from its list of disorders in 1973, with strong opposition from psychoanalysts. It took nearly three decades for the London-based International Psychoanalytical Association (IPA) to act similarly, in 2002. It seems likely that the IPA continues to tolerate the view that homosexuality is a disorder, treatable by psychoanalysis.^6,7^ The authors allude to ‘complex intrapsychic conflicts’ but fail to explain what they mean by this or provide a reference. This suggests an undeclared allegiance to psychoanalysis. Some British psychoanalysts appear to see transgender patients as a growth opportunity.^8^ The gender critical views of the ex-Tavistock London psychoanalyst Marcus Evans have been quoted by the BBC^9^ and the *BMJ*,^10^ while journalists have failed to scrutinise his implied claim that psychoanalysis can provide valid clinical opinion. Such scrutiny is especially necessary given the problematic relation of psychoanalysis to homosexuality and its wider history of evading scrutiny of its claims to therapeutic efficacy, validity^11^ and safety.^12^ Psychiatry should retain its gatekeeping role for transgender patients seeking physical treatments or legal gender change, but extreme gender critical views, which at present appear to include special pleading for psychoanalysis, would undermine the consent necessary for that role to be effective.
